# Characterization of Fecal Microbiota across Seven Chinese Ethnic Groups by Quantitative Polymerase Chain Reaction

**DOI:** 10.1371/journal.pone.0093631

**Published:** 2014-04-03

**Authors:** Lai-yu Kwok, Jiachao Zhang, Zhuang Guo, Qimu Gesudu, Yi Zheng, Jianmin Qiao, Dongxue Huo, Heping Zhang

**Affiliations:** Key Laboratory of Dairy Biotechnology and Bioengineering, Education Ministry of P. R. China, Department of Food Science and Engineering, Inner Mongolia Agricultural University, Hohhot, Inner Mongolia, P. R. China; Charité-University Medicine Berlin, Germany

## Abstract

The human gut microbiota consists of complex microbial communities, which possibly play crucial roles in physiological functioning and health maintenance. China has evolved into a multicultural society consisting of the major ethnic group, Han, and 55 official ethnic minority groups. Nowadays, these minority groups inhabit in different Chinese provinces and some of them still keep their unique culture and lifestyle. Currently, only limited data are available on the gut microbiota of these Chinese ethnic groups. In this study, 10 major fecal bacterial groups of 314 healthy individuals from 7 Chinese ethnic origins were enumerated by quantitative polymerase chain reaction. Our data confirmed that the selected bacterial groups were common to all 7 surveyed ethnicities, but the amount of the individual bacterial groups varied to different degree. By principal component and canonical variate analyses of the 314 individuals or the 91 Han subjects, no distinct group clustering pattern was observed. Nevertheless, weak differences were noted between the Han and Zhuang from other ethnic minority groups, and between the Heilongjiang Hans from those of the other provinces. Thus, our results suggest that the ethnic origin may contribute to shaping the human gut microbiota.

## Introduction

The human gut microbiota consists of complex communities of microorganisms. The roles of such communities are being increasingly recognized, and that these intact communities together act like a ‘microbial organ’ [1], [2], which may be involved in a number of physiological functions both directly and indirectly relating to digestion and metabolism, e.g. carbohydrate fermentation and absorption, energy acquisition, immunoregulation (recently reviewed by [3]–[5]). Current evidence also suggests a close relationship between the gut microbiota and various diseases or unhealthy scenarios including obesity, diabetes, allergies and rheumatoid arthritis [6]–[9]. Therefore, the balance of the gut microbiota composition appears to be crucial to the host health maintenance.

Traditionally, culture techniques have been used to study the human gut microbiota composition. However, one obvious problem of the currently available culture techniques is the high ‘unculturability’ of the vast diversity of gut microbes, which was predicted to be 20–80% [10]. Recent sequencing data indicate, on the other hand, that the fact that many gut bacteria remain uncultured may often be due to their low abundance rather than inherent unculturability [11]. Additionally, conventional culture techniques are time-consuming, laborious and, in many cases, produce ambiguous results. Therefore, more recent studies have turned into the molecular approach. For instance, studies using a combination of 16S rRNA gene PCR and pyrosequencing techniques have provided new insights into the gut microbiota composition and consistently revealed that *Firmicutes*, *Bacteroidetes*, *Actinobacteria* and *Proteobacteria* were the dominant phyla of the common core gut microbiota [12], [13]. Peris-Bondia et al. [14] pointed out that the three former phyla represented around 75% of the human gut microbial diversity.

Apart from characterizing the gut microbiota structure, understanding which factors shape the gut microbiota composition has become a recent focus. Controversial results have so far been reported. Cross-country surveys performed by Arumugam et al. [15] and Lay et al. [16] both showed no significant clustering of the gut microbiota diversity by the geographic origin of the subjects. Arumugam et al. [15] further suggested that the gut microbiota structure of healthy individuals was irrespective to host biometric factors such as age, body-mass index and gender. On the contrary, several other groups reported geographic-, ethnicity-, lifestyle- or diet-based clustering of the gut microbiota composition in similar type of studies [13], [17], [18]. The conflicting results observed from the various studies would need to be further clarified by a substantially broader cross-cultural sampling.

China has evolved into a multicultural society consisting of the major ethnic group, Han (91.65% of the Chinese population), and 55 official ethnic minority groups through thousand years of development. Representative minority groups include the Bai, Kazakh, Mongol, Tibetan, Uyghur and Zhuang. Nowadays, these minority groups inhabit in different provinces of China and some of them still keep their traditional culture, lifestyle, dietary habits and living environments. Such distinctive variations make China an attractive model for understanding how these factors affect the structure of the gut microbiota. Moreover, up to now, only limited data are available on the composition of the gut microbiota of these Chinese ethnic groups. Therefore, in this study, a large-scale nationwide survey was performed on the fecal microbiota of 314 healthy individuals of similar age and body mass index range. The studied subjects belonged to 7 different ethnic origins from 20 geographic locations, who adopted either an urban or rural lifestyle.

Although the 16S rRNA gene PCR in combination with pyrosequencing appears to be a successful approach in studying the microbial composition in an ecological environment like the human gut, it is limited to the estimation of the relative abundance but not absolute bacterial density [19]. Therefore, quantitative polymerase chain reaction (qPCR) has been suggested to provide more accurate quantitative information. In this study, we enumerated 10 selected groups of common core gut microbiota in the fecal samples of the Chinese healthy human subjects by qPCR. The generated data were analyzed by a principal component analysis-multivariate analysis of variance (PCA-MANOVA) approach. The study aims to find out whether the structure of fecal microbiota is stratified by factors like the ethnic origin, lifestyle, and geographic location of residence of the host. To our knowledge, this is the first large-scale survey providing detailed information on the fecal bacterial composition of Chinese individuals of different ethnicities.

## Materials and Methods

### Subjects

**Figure 1:**
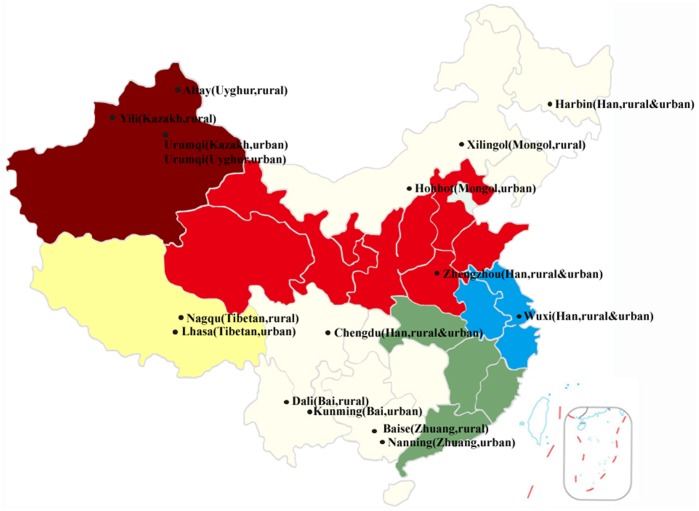
A map of sampling sites.

A total of 314 healthy individuals were recruited for the current study. Subjects were from 9 Chinese provinces belonging to 7 ethnic minority origins, who adopted either an urban or a rural-dwelling lifestyle. Participants were identified from the residential and university areas of the ethnic groups of interest within the chosen geographic regions. All of the studied individuals were indigenous residents who have been living in the locality for over three generations. To preclude selection bias, participants were solely identified based on age (18 to 35), body-mass index (18.5 to 24.9) and health conditions. The volunteers did not suffer from any gastrointestinal tract disorders or take any antibiotics for at least 3 months before the sampling time. No specific restriction was imposed on the socio-economic status or the dietary habits/intake of the participants in the selection process. The study protocol was approved by the Ethical Committee of the Inner Mongolia Agricultural University (Hohhot, China). After obtaining the written informed consent, a standard questionnaire was administrated to collect personal information of the subject. The sampling sites and information are summarized in Figure 1 and Table 1, respectively.

**Table 1:** A summary of sampling information.

	Sampling place				Individual ratio (*n*/*n*)	
Ethnic group	Province	Urban	Rural	Sample size (*n*)	Female/Male	Urban/Rural
Han	Heilongjiang	Nangang, Daoli and Daowai District of Harbin	Mulan and Tonghe County of Harbin	30	13/17	16/14
Han	Henan	Jinshui and Zhongyuan District of Zhengzhou	Dengfeng County of Zhengzhou	23	9/14	11/12
Han	Jiangsu	Chongan District of Wuxi	Binghu District of Wuxi	21	13/8	12/9
Han	Sichuan	Wuhou District of Chengdu	Dayi and Pujiang County of Chengdu	17	12/5	8/9
Zhuang	Guangxi	Xingning and Xixiangtang District of Nanning	Lingyun County of Baise	46	22/24	22/24
Uygur	Xinjiang	Shuimogou and Tianshan District of Urumqi	Altay County, Altay	21	10/11	12/9
Kazakh	Xinjiang	Shuimogou and Tianshan District of Urumqi	Yining and Tacheng County of Yili	22	13/9	13/9
Mongolian	Inner Mongolia	Saihan District of Hohhot	Bordered Yellow and Bordered White Banner of Xilinguole	48	25/23	22/26
Tibetan	Tibetan	Chengguan District of Lhasa	Nagqu County of Nagqu	43	21/22	15/28
Bai	Yunnan	Panlong and Xishan District of Kunming	Eryuan and Jianchuan County of Dali	43	19/24	14/29
**Total**				**314**	**157/157**	**145/169**

### Sample Collection, Transportation and Storage

Fecal samples were collected from each subject in the early morning before eating breakfast. Samples were stored anaerobically and were frozen immediately in liquid nitrogen before and during the transportation to the laboratory within 24 hours of collection. Samples were stored at −80°C until genomic DNA extraction.

### Genomic DNA Extraction

Genomic DNA was extracted from the samples within 24 hours of sample arrival to our laboratory according to methods described in Dethlefsen and Reman [20]. Briefly, samples were thawed on ice for 1 hour before genomic DNA extraction. They were homogenized by a bead-beating step in lysis buffer followed by a direct bacterial DNA extraction with the QIAamp DNA stool mini kit (Qiagen). The quality of the extracted genomic DNA was checked by agarose gel electrophoresis and spectrophotometric analysis. All DNA samples were stored at −20°C until further experiments.

### Enumeration of Target Bacteria Using qPCR

qPCR was used to quantify the total bacteria and the bacteria of interest in the stool samples. The amount of total bacteria was estimated by using the universal primers, Uni331F and Uni797R, which amplified a conserved region of the 16S rRNA for most common bacteria (Table 2). In order to ensure a broad bacterial coverage, representative dominant/subdominant groups from each of the four major phyla of gut microbiota were chosen as targets and were subjected to enumeration by qPCR with specific primers as listed in Table 2. The target bacterial groups included the *Clostridium coccoides* group, *Clostridium leptum* group, *Clostridium perfringens* group and *Lactobacillus* genus (*Firmicutes*), *Bacteroides fragilis* group and *Prevotella* genus (*Bacteroidetes*), *Bifidobacterium* genus and *Atopobium* cluster (*Actinobacteria*), *Enterobacteriaceae* family and *Desulfovibrio* genus (*Proteobacteria*).

**Table 2:** qPCR primer information and predicted product size.

Target bacterial group	Primers	DNA Sequence (5′- 3′)	Predicted PCR productsize (bp)	Annealingtemp (°C)	Reference
*Atopobium* cluster	Atopo-F	GGGTTGAGAGACCGACC	190	55	[38]
	Atopo-R	CGGRGCTTCTTCTGCAGG			
*Bacteroides fragilis* group	Bfra-F	ATAGCCTTTCGAAAGRAAGAT	495	50	[39]
	Bfra-R	CCAGTATCAACTGCAATTTTA			
*Bifidobacterium* genus	Bifid-F	CTCCTGGAAACGGGTGG	550	55	[39]
	Bifid-R	GGTGTTCTTCCCGATATCTACA			
*Clostridium coccoides* group	Ccoc-F	AAATGACGGTACCTGACTAA	440	50	[39]
	Ccoc-R	CTTTGAGTTTCATTCTTGCGAA			
*Clostridium leptum group*	sg-Clept-F	GCACAAGCAGTGGAGT	239	55	[38]
	sg-Clept-R3	CTTCCTCCGTTTTGTCAA			
*Clostridium perfringens group*	ClPER-F	AGATGGCATCATCATTCAAC	170	60	[40]
	ClPER-R	GCAAGGGATGTCAAGTGT			
*Desulfovibrio* genus	Dsv-F	CCGTAGATATCTGGAGGAACATCAG	135	63	[41]
	Dsv-R	ACATCTAGCATCCATCGTTTACAGC			
*Enterobacteriaceae* family	Enter-F	TGCCGTAACTTCGGGAGAAGGCA	428	60	[42]
	Enter-R	TCAAGGACCAGTGTTCAGTGTC			
*Lactobacillus* genus	Lac-F	AGCAGTAGGGAATCTTCCA	341	58	[43]
	Lac-R	CACCGCTACACATGGAG			
*Prevotella* genus	g-Prevo-F	CACRGTAAACGATGGATGCC	528	55	[38]
	g-Prevo-R	GGTCGGGTTGCAGACC			
All bacteria	Uni331F	TCCTACGGGAGGCAGCAGT	466	58	[44]
	Uni797R	GGACTACCAGGGTATCTATCCTGTT			

qPCR was performed in an ABI Step-One detection system (Applied Biosystems, Inc.) using SYBR Premix Ex *Taq* II kits (TaKaRa Bio Inc.). To optimize the assay, 5 different genomic DNA template amounts (ranging from 0.01 to 100 ng) were tested to ensure that the qPCR was not suppressed in case any inhibitor was co-extracted with the genomic DNA. Both of the highest amounts (10 and 100 ng) that we tested were not inhibitory to the qPCR, as judging from the qPCR amplification curves (data not shown). In order to maximize the amplification of the relatively less abundant bacterial target groups, the amount of 100 ng was used in each reaction. Each reaction was run in duplicate in a final volume of 20 μL, consisting of 10 μL of 2x SYBR Premix Ex *Taq* II, 0.2 mM final concentration of each primer, a fixed amount of 100 ng of genomic DNA and an appropriate amount of deionized water. The amplification program consisted of 1 cycle of 95°C for 20 s, followed by 40 cycles of 95°C for 5 s, appropriate annealing temperature (Table 2) for 30 s and 72°C for 35 s, and finally 1 cycle of 94°C for 15 s. The fluorescent products were detected at the last step of each cycle.

### Bacterial Strains and Standard Curves for qPCR

Bacterial strains used for standard curve construction, the efficiency and coefficient of determination (R_2_ value) of qPCR are listed in Table 3. Culture conditions used for growing the standard bacterial strains are described in Table S1. For quantifying each bacterial group, a standard curve was constructed with the respective reference strain. qPCR was performed as described above with serially diluted bacterial genomic DNA extracted from a known amount of cells using the QIAamp genomic DNA kit (Qiagen), and the respective copy number of 16S rRNA was calculated. The respective standard curve was constructed by plotting threshold cycles (Ct) against bacterial quantity (in 16S rRNA). The bacterial amounts in the samples were determined by interpolating from the generated standard curves.

**Table 3:** Bacterial strains used for construction of standard curves, efficiency and coefficient of determination of qPCR.

Target bacterial group	Standard strain and strain number	qPCR efficiency (%)	Coefficient of determination (R)^2^
*Atopobium* cluster	*Atopobium parvulum* JCM 10300	101.5	0.992
*Bacteroides fragilis* group	*Bacteroides fragilis* ATCC 25285	102.4	0.996
*Bifidobacterium* genus	*Bifidobacterium breve* ATCC 15700	98.5	0.998
*Clostridium coccoides* group	*Blautia coccoides* JCM 1395	98.3	0.999
*Clostridium leptum* group	*Clostridium leptum* DSM 753	97.6	0.991
*Clostridium perfringens* group	*Clostridium perfringens* ATCC 13124	95.4	0.995
*Desulfovibrio* genus	*Desulfovibrio desulfuricans* ATCC 13541	99.2	0.995
*Enterobacteriaceae* family	*Escherichia coli* ATCC 11775	95.8	0.998
*Lactobacillus* genus	*Lactobacillus casei* ATCC 393	93.5	0.998
*Prevotella* genus	*Prevotella oralis* JCM 6330	96.2	0.997
All bacteria	*Lactobacillus casei* ATCC 393	97.6	0.992

### Statistical Analyses

Bacterial amounts were expressed as mean values ± standard error. Mann-Whitney test with Bonferroni correction for multiple testing was used to evaluate the sample difference in a pairwise manner. Two sample groups with a corrected p-value of less than 0.05 were considered significantly different from each other. Data of bacterial amounts and fecal *Firmicutes*/*Bacteroidetes* ratios of different ethnic groups were presented as box-plots. PCA, MANOVA and cluster analysis were used to display any clustering pattern/tendency of sample bacterial composition. All the statistical analyses were performed in Matlab R2011b (The MathWorks, Natick, MA, USA), with the PAST software [21] and the online statistical tools developed by Kirkman [22].

## Results

### Relative Abundance of the Target Bacterial Groups

Fecal samples were subjected to qPCR. In order to ensure a broad coverage of members of fecal bacteria, 10 major bacterial groups from the 4 dominant phyla of the common core gut microbiota were targeted. The relative abundance of the target bacterial groups is expressed in ratio of the specific group to ‘all bacteria’ (Figure S1). Significant variation was only observed between the Mongolians and the Han, Bai and Zhuang subjects (p = 0.0311, 0.0100 and 0.0454, respectively).

### Comparison of Fecal Bacteria of Different Ethnic Groups

**Figure 2:**
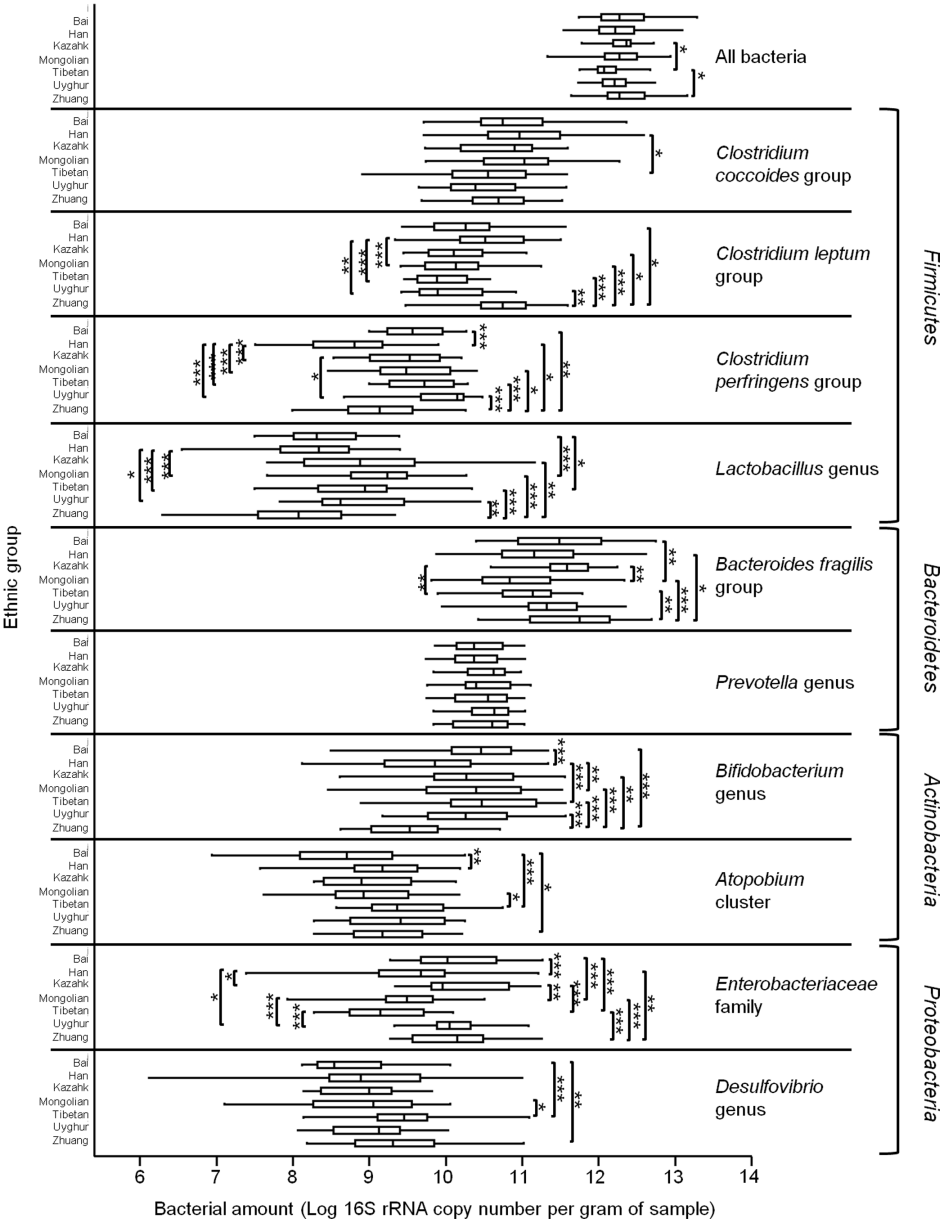
Box plots showing fecal bacterial composition of different ethnic groups. Bacterial amounts are expressed in Log 16S rRNA copy number per gram of fecal sample. Boxes show the median, 25^th^ and 75^th^ percentiles. The lower and upper adjacent hinges show the minimum and maximum values. The significant difference between sample pair was evaluated by pairwise Mann-Whitney test with Bonferroni correction. A corrected p-value <0.05, 0.01 and 0.001 are denoted by ‘*’, ‘**’, and ‘***’, respectively. The obtained p-values are given in Table S3.

The composition of fecal bacteria in different ethnic groups is presented in Figure 2 and Table S2. The amount of ‘all bacteria’ detected by the universal primers used in this study revealed no significant difference between most ethnic groups except that Tibetan<Kazahk, Zhuang (p = 0.0108 and 0.0140, respectively) (Table S3). The sum amount of the 10 target bacterial groups was lower in the Tibetan than most other ethnic groups (Tibetan<Kazahk, Zhuang and Bai, p = 0.0011, 0.0041 and 0.0018, respectively) (Table S3).

The 10 bacterial groups exhibited different degree of variation across the 7 ethnic groups (Figure 2). For each bacterial group, 21 pairwise Mann-Whitney comparison was performed (corrected p-values are shown in Table S3). The least variable bacterial groups which showed only 0 to 4 significantly differing pairs were the *Prevotella* genus (no significant difference across the 7 ethnic groups), *Clostridium coccoides* group (Tibetan<Han, p = 0.0104), *Desulfovibrio* genus (Bai, Mongolian<Tibetan, Bai<Zhuang; p = 0.0001–0.0423) and *Atopobium* cluster (Bai<Han, Tibetan and Zhuang, Mongolian<Tibetan; p = 0.0003–0.0275). 6 to 11 significantly differing pairs were found in the *Bifidobacterium* genus (Zhuang<Bai, Mongolian, Tibetan, Kazahk and Uyghur, Han<Bai, Mongolian and Tibetan; p = 0.0000–0.0027), *Lactobacillus* genus (Zhuang< Kazahk, Mongolian, Tibetan and Uyghur, Han<Mongolian, Tibetan and Uyghur, Bai<Mongolian and Tibetan; p = 0.0000–0.0242), *Clostridium leptum* group (Zhuang>Bai, Mongolian and Tibetan, Kazahk and Uyghur, Han>Mongolian, Tibetan and Uyghur; p = 0.0000–0.0317), *Bacteroides fragilis* group (Zhuang>Han, Mongolian and Tibetan; Mongolian and Tibetan> Kazahk; Mongolian>Bai; p = 0.0001–0.0245), and *Clostridium perfringens* group (Han>all other ethnic groups, Zhuang>all other ethnic groups except for Han; p = 0.0000–0.0432). The most variable group was the *Enterobacteriaceae* family showing 12 significantly differing pairs (Bai, Kazahk, Uyghur and Zhuang>Han, Mongolian and Tibetan; p = 0.0000–0.0253).

### Comparison of Fecal Bacteria of Subjects Living Different Lifestyle

The amount of each target bacterial group was compared between the urban- and rural-dwelling subgroups within each ethnic group using pairwise Mann-Whitney test. No significantly differing sample pair was identified, and indeed the majority of the sample pairs yielded a p-value of 1.0000 after Bonferroni correction for multiple testing. The only exceptions were observed in the *Clostridium perfringens* group (p = 0.0605) and the *Bacteroides fragilis* group (p = 0.0879) of the Mongolians (data not shown).

### Multivariate Analysis of Bacterial Composition with Ethnicity

**Figure 3:**
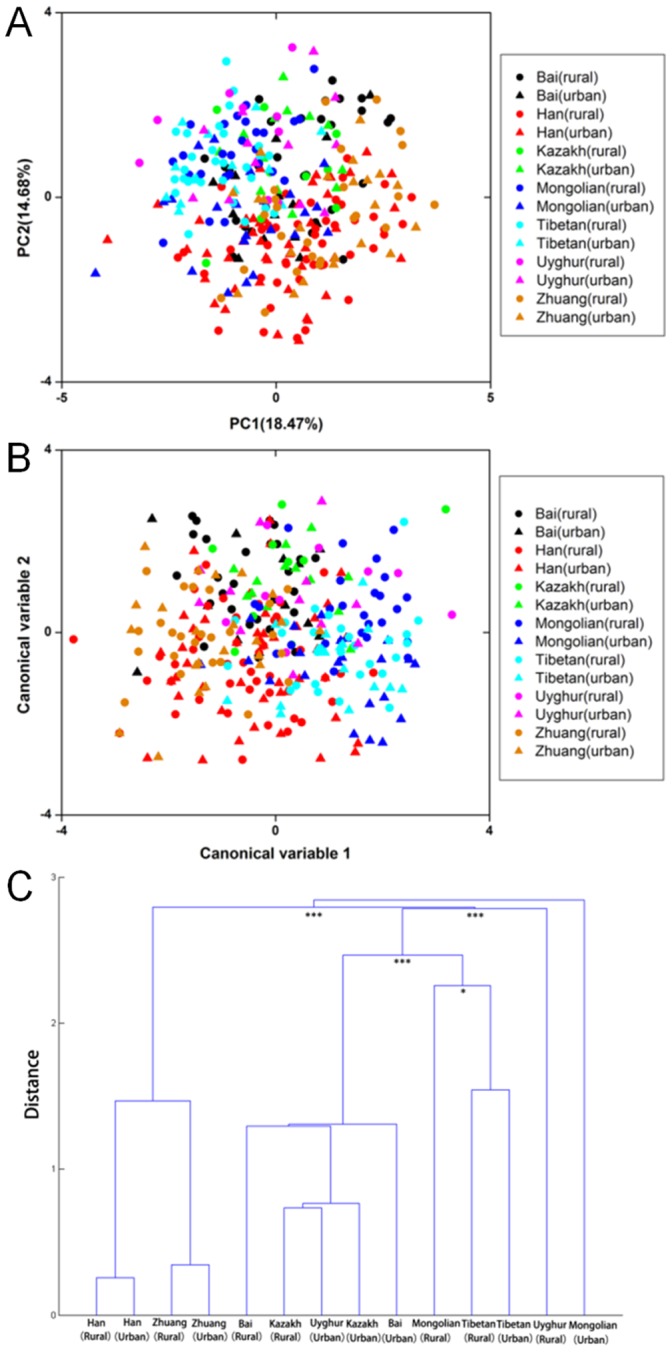
Variation of the fecal bacterial composition across the 7 ethnic groups. A. PCA score plot of the first two principal components with the proportion of variance explained by each component printed next to the axes labels. B. MANOVA score plot using the first 9 principal components. Individuals are classified according to their corresponding ethnic group and lifestyle. The first two canonical variables are plotted. Each individual is represented by one dot and the color label corresponds to the ethnic origin and lifestyle. C. Dendrogram constructed based on the distance metrics of different ethnic groups. ‘*’ and ‘***’ indicate p<0.05, 0.001, respectively.

PCA was used to display any clustering pattern of fecal bacteria of the 314 individuals. No distinct grouping was observed within any ethnic group (Figure 3A). MANOVA was further performed to analyze the variability of the 314 samples. Similarly, no distinct grouping was observed. However, there was a tendency of accumulation of the Tibetan and Mongolian samples at the right quadrants of the score plot, whereas most of the Zhuang and Han samples were located at the left quadrants (Figure 3B), suggesting a mild difference of the Tibetan and Mongolian samples from those of the Zhuang and Han. Results from the cluster analysis supported such difference (Figure 3C and Figure S2). Samples from the Han and Zhuang formed a distinct cluster separated from the other ethnic groups. The Bai, Kazakh and Uyghur (urban-dwelling) grouped together. The Tibetan and Mongolian (rural-dwelling) were closer to each other than to other ethnic groups.

### Comparison of Fecal Bacteria of Han Individuals from 4 Different Provinces

The amounts of fecal bacteria from the Han subjects of the 4 Chinese provinces, Jiangsu, Sichuan, Henan and Heilongjiang, are shown in Table S4. For each bacterial group, 6 pairwise comparisons were made between the 4 provinces. Out of the 60 possible combinations for all 10 bacterial targets, only 6 were of significant difference and 5 of which were attributed to the Heilongjiang province (Jiangsu>Henan and Heilongjiang in *Desulfovibrio* genus, Heilongjiang>all 3 other provinces in *Clostridium perfringens* group, Sichuan>Heilongjiang in *Bacteroides fragilis* group; p = 0.0000–0.0249) (Table 4).

**Table 4:** Pairs of Han subgroups showing significant difference in bacterial quantity.

Sample pair showing significant difference	Bacterial family	Bonferroni-corrected p-value
Jiangsu - Henan	*Desulfovibrio* genus	0.0060
Heilongjiang - Jiangsu	*Desulfovibrio* genus	0.0027
Heilongjiang - Henan	*Clostridium perfringens* group	0.0098
Heilongjiang - Sichuan	*Clostridium perfringens* group	0.0002
Heilongjiang - Jiangsu	*Clostridium perfringens* group	0.0000
Heilongjiang - Sichuan	*Bacteroides fragilis* group	0.0249

Remark: Pairwise Mann-Whitney test was performed to evaluate the sample difference.

### Multivariate Analysis of Bacterial Composition with Geographic Data of the Han

**Figure 4:**
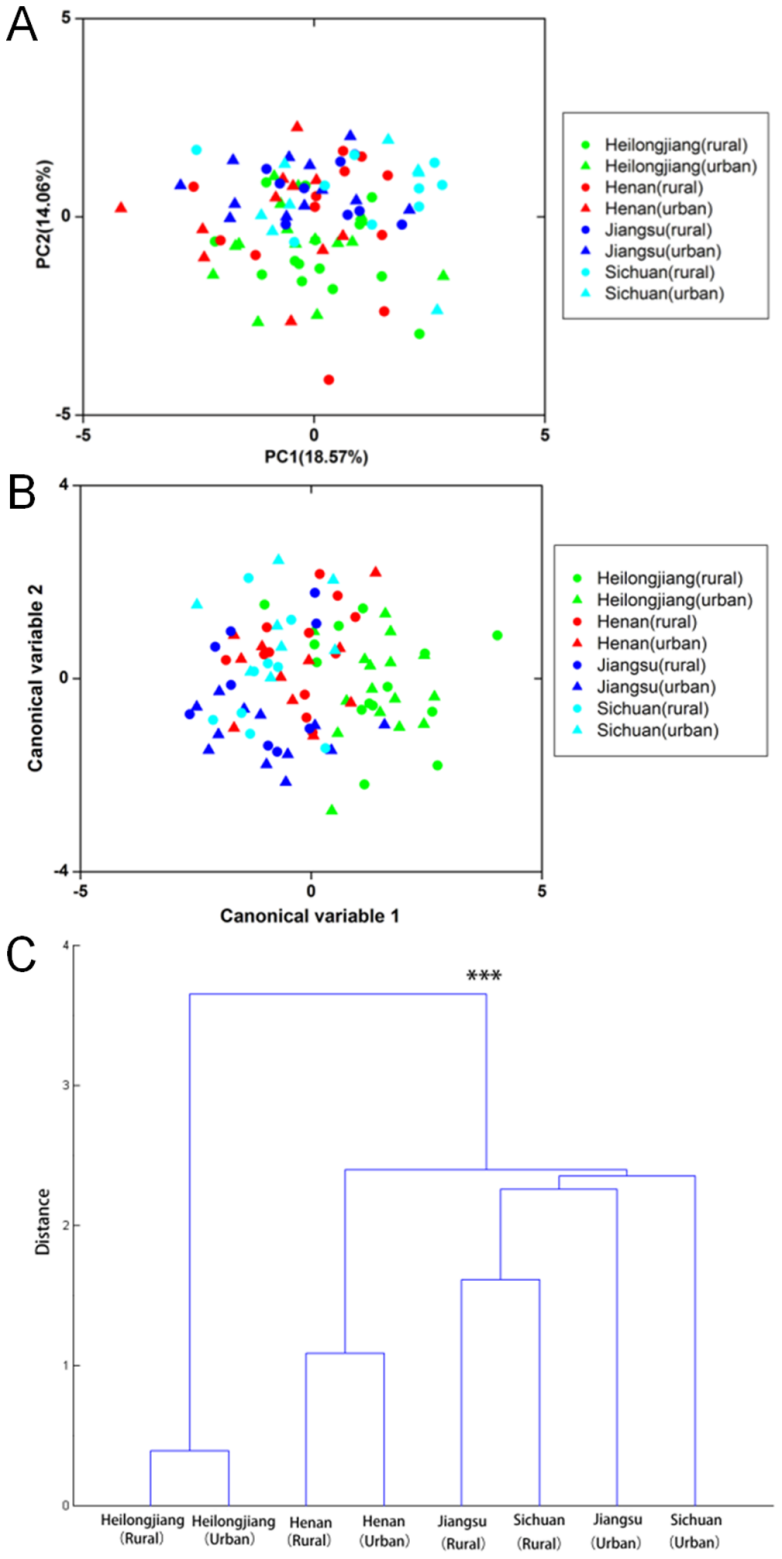
Variation of fecal bacterial composition of 91 Han subjects from 4 different provinces. A. PCA score plot. The two first principal components are plotted with the proportion of variance explained by each component printed next to the axes labels. B. MANOVA score plot using the first 9 principal components. Individuals are classified according to their province of origin and lifestyle. The first two canonical variables are plotted. Each individual is represented by one dot and the color label corresponds to the province of origin and lifestyle. C. Dendrogram constructed based on the distance metrics of Han subgroups from the 4 different provinces living an urban or rural lifestyle. ‘*’, ‘**’ and ‘***’ indicates p<0.05, 0.01 and 0.001, respectively.

No distinct group clustering was observed between the Han individuals from the 4 different Chinese provinces by PCA and MANOVA (Figures 4A and 4B). However, there was only a slight overlapping between the samples of the Heilongjiang Han with those from other provinces on both the PCA and MANOVA score plots, indicating a weak difference of the Heilongjiang Han samples from the others. This result was in agreement with the cluster analysis (Figure 4C).

### Comparison of *Firmicutes*/*Bacteroidetes* (*F*/*B*) Ratio in Different Ethnic Groups

**Figure 5:**
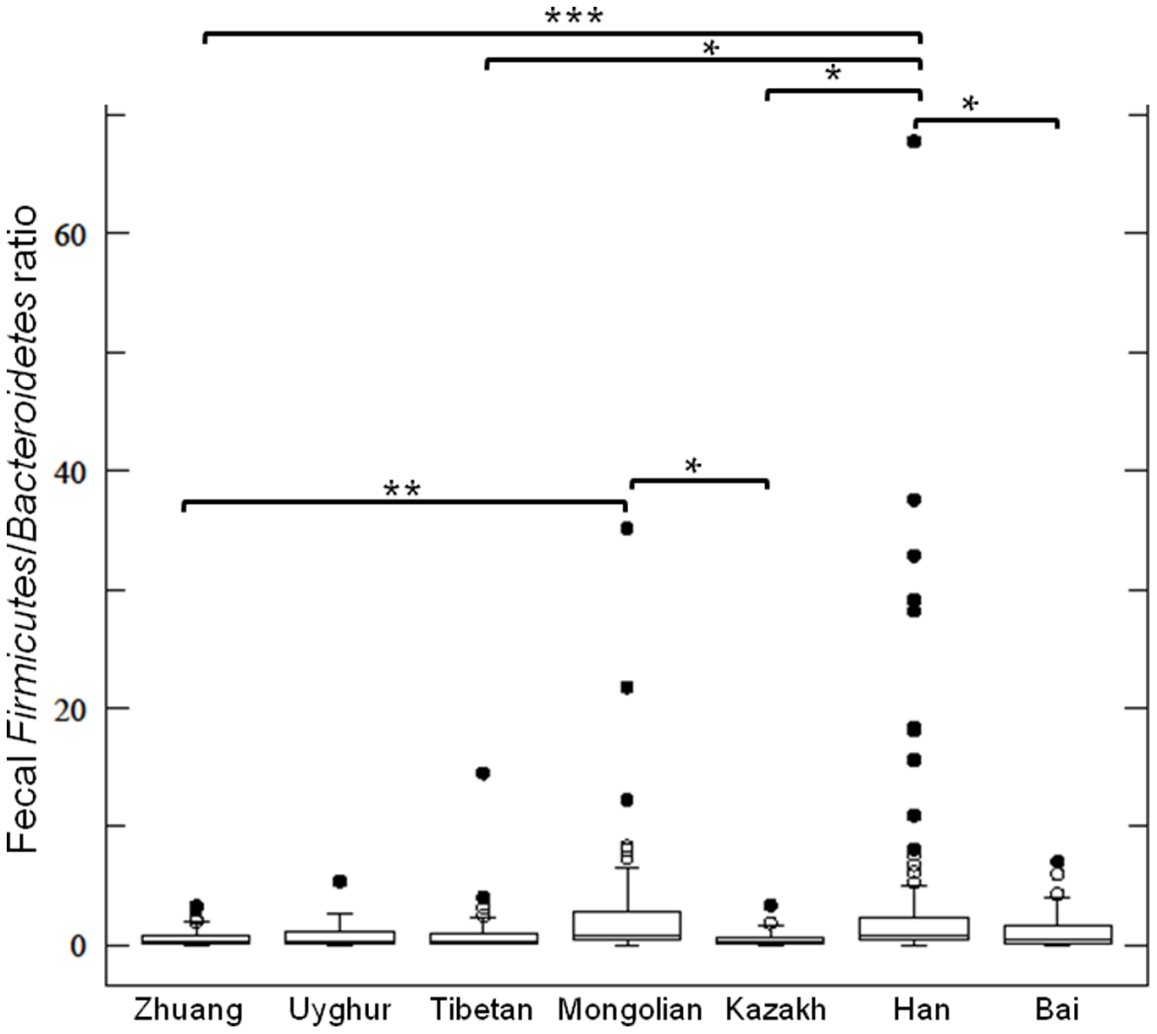
Box-plots of the *Firmicutes*/*Bacteroidetes* ratio of the 7 ethnic groups. Boxes show the median, 25^th^ and 75^th^ percentiles. The lower and upper adjacent hinges are 1.5× interquartile range (IQR) from the 1^st^ and 3^rd^ quartiles, respectively. ‘Suspected upper outliers’ >1.5 and >3 times of the IQR above the 3^rd^ quartile are symbolized with open and filled circles, respectively. No lower outlier was detected in any of the groups. The significant difference between sample pair was evaluated by pairwise Mann-Whitney test with Bonferroni correction. A corrected p-value <0.05 and 0.01 are denoted by ‘*’ and ‘**’, respectively. The obtained p-values are given in Table S5.

The *F*/*B* ratio of each ethnic group was calculated based on the quantities of the target bacterial groups determined in this study (Figure 5 and Table S5). Samples from the Han subjects had a significantly higher *F*/*B* ratio than most other surveyed ethnic groups, including the Zhuang, Tibetan, Bai and Kazakh (4.03±1.03 in Han, ranging from 0.56±0.16 to 1.08±0.35 in other groups, p = 0.0005 to 0.0465). The overall *F*/*B* ratio of the Mongolian samples was also generally higher than that of other surveyed ethnic groups except for Han.

## Discussion

Several ethnic- and geographic-based surveys were previously performed in the US, European and Korean populations 13,15–18,23, which suggested that the host ethnic origin/genetic background, geographic location of residence, lifestyle, diets, and age were potential factors that play key roles in shaping the fecal microbial composition. However, which are the dominating regulatory factors remain controversial. In this study, we took the advantage of the enormous country size, the wide distribution of the Han group and the presence of multiple ethnic minority groups in China, and performed an ethnic- and geographic-based preliminary survey on the fecal bacterial composition across 7 Chinese ethnic groups.

We confirmed that the 10 selected groups of bacteria were part of the common core fecal microbiota shared by the 7 surveyed ethnic groups, but the amount of the individual bacterial groups varied to different extent. Most likely, owing to the high commonality of the selected target bacterial groups across all the surveyed ethnic populations, the clustering pattern of the sample groups was not distinct on the PCA and MANOVA score plots. Nevertheless, several interesting trends were observed and were supported by our cluster analysis.

Firstly, the Han and Zhuang groups shared relatively high similarity. Samples from both the Han and Zhuang groups contained a higher amount of the *Clostridium leptum* group and less of the *Clostridium perfringens* group, *Lactobacillus* and *Bifidobacterium* genera, as compared to most other ethnic minority groups. Our cluster analysis showed that most of the Mongolian and the Tibetan samples grouped together, so as most of those from the Kazakh, Uyghur and Bai individuals. We found that such grouping pattern is in good agreement with the genetic clustering between these ethnic groups.

By genetic analyses, Zhuang was found to group with the southern Han and some other southern tribes, whereas the Tibetan, Mongolian, Uyghur and Kazahk clustered with other northern tribes [24], [25]. Owing to the migratory history of these tribes, the genetic clustering pattern of the Chinese ethnic minority groups is not strictly correlated with their current geographic location of residence. The Chinese Uyghur and Kazahk both live in the Xinjiang province, which was historically the Silk Road region in China. These two tribes, but not the Han living in the same area, were found to have high frequencies of western Eurasian-specific haplogroup, suggesting an extensive admixture of these two ethnic minority groups and their intrinsic genetic difference from the Han, and from other northern groups [26]. Both the Bai and Zhuang reside in the southwest Chinese provinces; surprisingly the Bai was found to cluster with the Uyghur and Kazahk instead of the Zhuang from our results. This may be explained by the distant genetic relationship between the Bai and Zhuang, which failed to cluster together by microsatellite marker analysis [27]. The genetic difference between these two tribes can be traced back to their distinct ancestral origins. Interestingly, an ethnic-based analysis of the mitochondrial DNA sequence diversity revealed that the Bai had a slightly shorter genetic distance to the Uyghur than to the Zhuang [25].

Our second interesting observation was the difference between the Hans from Heilongjiang, the northernmost Chinese province, from those residing in the centrally located Henan, Jiangsu and Sichuan provinces. There is strong evidence supporting the genetic segregation of ‘southern’ and ‘northern’ Hans as revealed by previous analyses of Y-chromosome markers, mitochrondrial DNA and single nucleotide polymorphism of Han subjects across the country [28]–[30]. It appears that both the geographic and genetic distances are possible factors relating to the stratification of the gut microbiota.

Thirdly, lifestyle did not seem to be a dominating factor in modulating the gut microbiota, as none of the urban or rural lifestyle subgroups from the same ethnic tribe showed a significant difference in any of the target bacterial groups. Although we did not impose any specific restriction on the social-economic status during the subject selection, most of our rural area subjects lived either on farms (Han, Bai, Zhuang and Uyghur) or the traditional nomadic lifestyle (Tibetan, Mongolian and Kazahk), contrasting to the diversified background of the urban area subjects. No tendency of clustering of the rural area subgroups was observed. Additionally, the long history of multi-ethnicity in China has resulted in different extent of Han assimilation. The Tibetan, Mongolian and Uyghur are rather resistant in assimilating into the Han’s culture and lifestyle compared to the Zhuang and Bai [31]. It is hard to explain purely from the perspective of lifestyle why the Bai group clustered with the Kazakh and Uyghur, but not with the Han and Zhuang.

Diet intake is thought to be another important factor that shapes the gut microbiota. One limitation of the current study is the lack of dietary information on the subjects. However, previous large-scale surveys revealed the existence of obvious differences in dietary intake between the Chinese ethnic minority groups [32], [33]. For example, the Tibetan and Kazakh live in highlands and mountainous areas, which make vegetables less accessible to them, and, hence, their diets are consisted of low fiber. Ethnic groups residing in southern China generally have a higher consumption of vegetables. For instance, the Zhuang consumed a double daily amount of vegetables compared to the Tibetan and Kazakh and a much higher amount of legumes than other studied ethnic minority groups, although they also had a high daily intake of meat [32], [33]. Filippo et al. [17] suggested that the daily intake of rich-fiber diets would enrich the fecal *Bacteroidetes*, in particular the *Prevotella* genus, and diminish the *Firmicutes*. Contrarily, neither the proportion of *Firmicutes* or *Bacteroidetes* was significantly different between the Tibetan/Kazakh and the Zhuang, nor any variation in the amount of the *Prevotella* genus was found across the 7 ethnic groups even though their dietary difference. The Uyghur and Kazahk exhibited a high level of commonality in their diet, including a high daily intake of dairy products and salt [32], which might have supported their clustering tendency seen in our results.

Mariat et al. [34] reported the correlation of age with the fecal *F*/*B* ratio (0.4, 10.9 and 0.6 for infants, adults and elderly individuals, respectively). We performed similar analysis and found that the Han had a significantly higher *F*/*B* ratio than the Zhuang, Tibetan, Bai and Kazakh, though the difference was far less striking than that observed by Mariat et al. [34] (4.03±1.03 in Han, ranging from 0.56±0.16 to 1.08±0.35 in the other four ethnic groups, p = 0.0005 to 0.0465). Similarly, the Mongolians had an overall higher *F*/*B* ratio than other ethnic groups. Moreover, both the Han and Mongolian subjects seemed to have a higher proportion of suspected outlier data points (14.29% and 12.59, respectively) compared to the other studied ethnic groups (ranging from 4.76–9.30%). A decrease in the *Bifidobacterium* genus and an increase in the *Enterobacteriaceae* family were known to be accompanied by the ageing process [35]. Our results revealed that the fecal samples of the Han and Zhuang had lower amount of *Bifidobacterium*, whereas the Tibetan ones had lower amount of *Enterobacteriaceae* than most other surveyed ethnic groups. Since all our volunteers were within a narrow age range (between 18 to 35 years old), our results suggested that some of these previously defined ageing-related fecal microbial differences might also be partly related to the host ethnic origin.

The present study used qPCR to quantify some of the dominant/subdominant gut bacterial groups. The qPCR approach was advantageous over the recently developed 16S rRNA-pyrosequencing approach, as it is economical, easy to perform, and, more importantly, provide more accurate quantitative data. However, the current approach does suffer from several limitations. Although the major dominant and subdominant bacterial groups were targeted in our study, the bacterial coverage was still incomplete. Technically, it is infeasible to detect ‘all target bacteria’ in the reaction, and the variation of efficiency of the individual qPCR might result in changing the overall proportion of the various bacterial groups. These factors may contribute to variation of results and cause difficulty in direct comparison between studies.

Nevertheless, there are large variations of the reported data in the literature, which can be related to the natural inter-individual variations and/or other technical reasons, including the choice of methods (e.g. pyrosequencing, HITchip, 16S rRNA clone libraries versus qPCR), PCR and sequencing primers, and specific experimental steps. Sample storage and DNA extraction methods are also known to influence the results of microbial community studies and have led to contradictory inferences [36], [37]. Without a direct experimental comparison of samples, it is, therefore, hard to distinguish whether any difference between studies is due to the laboratory procedures, data handling or truly attributed to the samples.

## Conclusions

The current study has provided preliminary and comparative information on the abundance of some major fecal bacterial groups across 7 Chinese ethnic groups. The vast amount of current literature in the field together suggests that the gut microbiota structure is most likely modulated by a combination of host factors and environmental exposures. Our experimental design did not single out any of these factors, and therefore we cannot exclude the significance of any of them. However, the host genetic background seems to be the factor that best explains our current observations, thus the ethnic origin does appear to play an important role in shaping the human gut microbiota.

## Supporting Information

Figure S1 **Ratio of target bacterial groups to ‘all bacteria’ in the 7 ethnic groups.**
(TIF)

Figure S2 **Dendrogram constructed based on the distance metrics of different ethnic groups.** ‘*’ and ‘***’ indicate p<0.05, 0.001, respectively.
(TIF)

Table S1 **Culture conditions for standard bacterial strains.**
(DOC)

Table S2 **Bacterial amounts of the fecal samples of 7 ethnic groups.**
(DOC)

Table S3 **Bonferroni-corrected p-values generated by pairwise Mann-Whitney test comparing bacterial amounts of fecal samples from 7 ethnic groups.**
(DOC)

Table S4 **Bacterial amounts of the 91 fecal samples of the Han subjects.**
(DOC)

Table S5 ***Firmicutes*** **/** ***Bacteroidetes*** **(** ***F/B*** **) ratio of different ethnic groups.**
(DOC)
