# Supplementary figures and images for: Characterization of Fecal Microbiota across Seven Chinese Ethnic Groups by Quantitative Polymerase Chain Reaction
Source: PLoS One. 2014 Apr 3;9(4):e93631. doi: 10.1371/journal.pone.0093631 (PMC3974763; doi:10.1371/journal.pone.0093631)

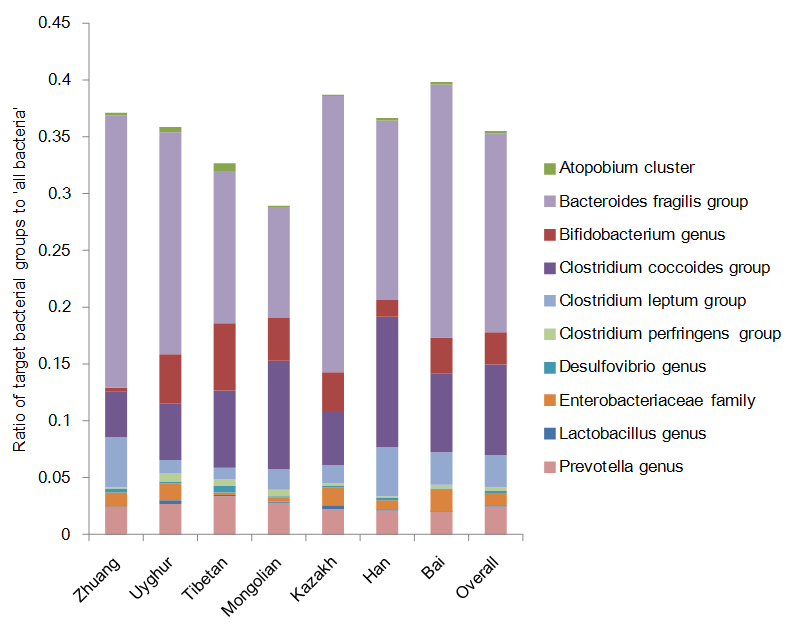

Supplement: Figure S1 — Ratio of target bacterial groups to ‘all bacteria’ in the 7 ethnic groups. (TIF) [file pone.0093631.s001.tif]

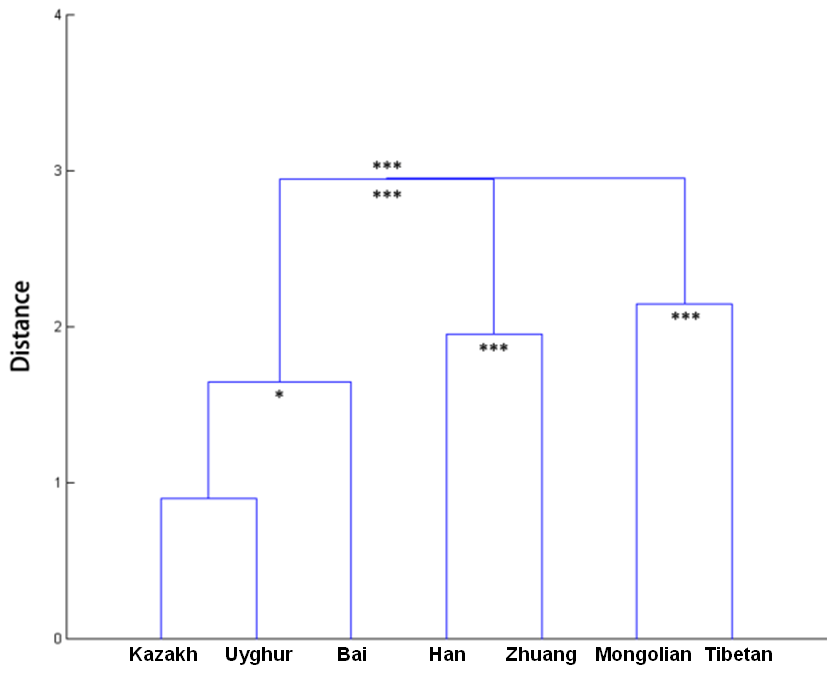

Supplement: Figure S2 — Dendrogram constructed based on the distance metrics of different ethnic groups. ‘*’ and ‘***’ indicate p<0.05, 0.001, respectively. (TIF) [file pone.0093631.s002.tif]
