# Supplementary material for: Characterization of Fecal Microbiota across Seven Chinese Ethnic Groups by Quantitative Polymerase Chain Reaction
Source: PLoS One. 2014 Apr 3;9(4):e93631. doi: 10.1371/journal.pone.0093631 (PMC3974763; doi:10.1371/journal.pone.0093631)
Supplement: Table S1 — Culture conditions for standard bacterial strains. (DOC) [file pone.0093631.s003.doc]

Table S1. **Culture conditions for standard bacterial strains**

| Standard bacterial strains | Culture media | Culture conditions | Cultivation temperature |
| --- | --- | --- | --- |
| *Atopobium parvulum* JCM 10300 | EG MEDIUM (JCM Medium 14) | Anaerobic | 37℃ |
| *Bacteroides fragilis* ATCC 25285 | EG MEDIUM (JCM Medium 14) | Anaerobic | 37℃ |
| *Bifidobacterium breve* ATCC 15700 | BL AGAR (JCM Medium 13) | Anaerobic | 37℃ |
| *Blautia coccoides* JCM 1395 | EG MEDIUM (JCM Medium 14) | Anaerobic | 37℃ |
| *Clostridium leptum* DSM 753 | EG MEDIUM (JCM Medium 14) | Anaerobic | 37℃ |
| *Clostridium perfringens* ATCC 13124 | EG MEDIUM (JCM Medium 14) | Anaerobic | 37℃ |
| *Desulfovibrio desulfuricans* ATCC 13541 | DVO5 MEDIUM (JCM Medium 439) | Anaerobic | 30℃ |
| *Escherichia coli* ATCC 11775 | Nutrient agar | Atmospheric conditions | 37℃ |
| *Lactobacillus casei* ATCC 393 | MRS agar | Atmospheric conditions supplemented with 5% CO_2_ | 37℃ |
| *Prevotella oralis* JCM 6330 | EG MEDIUM (JCM Medium 14) | Anaerobic | 37℃ |

Remarks: For anaerobic cultivation, bacteria were grown at standard anaerobic chamber of 80% N_2_, 10% CO_2_, and 10% H_2_.

JCM stands for Japan Collection of Microorganism.
