# Supplementary material for: Characterization of Fecal Microbiota across Seven Chinese Ethnic Groups by Quantitative Polymerase Chain Reaction
Source: PLoS One. 2014 Apr 3;9(4):e93631. doi: 10.1371/journal.pone.0093631 (PMC3974763; doi:10.1371/journal.pone.0093631)
Supplement: Table S2 — Bacterial amounts of the fecal samples of 7 ethnic groups. (DOC) [file pone.0093631.s004.doc]

Table S2. **Bacterial amounts of the fecal samples of 7 ethnic groups**

|  |  | *Firmicutes* | | | | *Bacteroidetes* | | *Actinobacteria* | | *Proteobacteria* | |  |
| --- | --- | --- | --- | --- | --- | --- | --- | --- | --- | --- | --- | --- |
|  | All bacteria | *Clostridium coccoides* group | *Clostridium leptum* group | *Clostridium perfringens* group | *Lactobacillus* genus | *Bacteroides fragilis* group | *Prevotella* genus | *Bifidobacterium* genus | *Atopobium* cluster | *Enterobacteriaceae* family | *Desulfovibrio* genus | Sum of the 10 bacterial groups |
| Zhuang-A | 12.34±0.05 | 10.69±0.06 | 10.66±0.08 | 9.14±0.09 | 8.07±0.10 | 11.60±0.09 | 10.49±0.06 | 9.49±0.08 | 9.23±0.08 | 10.06±0.08 | 9.35±0.10 | 11.88±0.06 |
| Zhuang-U | 12.34±0.07 | 10.81±0.09 | 10.82±0.12 | 9.20±0.13 | 8.16±0.16 | 11.61±0.12 | 10.40±0.09 | 9.50±0.13 | 9.31±0.12 | 10.05±0.13 | 9.33±0.14 | 11.92±0.08 |
| Zhuang-R | 12.33±0.08 | 10.58±0.09 | 10.51±0.11 | 9.10±0.12 | 8.00±0.13 | 11.58±0.14 | 10.57±0.08 | 9.47±0.11 | 9.16±0.10 | 10.07±0.10 | 9.37±0.15 | 11.85±0.09 |
| Uyghur-A | 12.22±0.06 | 10.52±0.13 | 10.03±0.10 | 9.92±0.11 | 8.90±0.16 | 11.35±0.13 | 10.58±0.08 | 10.29±0.14 | 9.37±0.15 | 10.09±0.11 | 9.01±0.13 | 11.75±0.06 |
| Uyghur-U | 12.27±0.08 | 10.68±0.18 | 10.03±0.14 | 9.84±0.13 | 8.82±0.23 | 11.33±0.17 | 10.65±0.09 | 10.16±0.18 | 9.08±0.17 | 10.15±0.16 | 9.12±0.17 | 11.75±0.09 |
| Uyghur-R | 12.16±0.10 | 10.31±0.16 | 10.02±0.14 | 10.03±0.17 | 9.02±0.23 | 11.37±0.22 | 10.48±0.13 | 10.47±0.22 | 9.75±0.19 | 10.02±0.14 | 8.87±0.20 | 11.75±0.10 |
| Tibetan-A | 12.10±0.03 | 10.57±0.09 | 9.95±0.06 | 9.66±0.06 | 8.83±0.09 | 11.07±0.07 | 10.46±0.06 | 10.53±0.10 | 9.50±0.09 | 9.22±0.08 | 9.43±0.09 | 11.57±0.03 |
| Tibetan-U | 12.12±0.06 | 10.50±0.18 | 9.99±0.08 | 9.81±0.10 | 8.68±0.15 | 11.09±0.15 | 10.39±0.10 | 10.31±0.18 | 9.65±0.18 | 9.18±0.14 | 9.35±0.13 | 11.55±0.07 |
| Tibetan-R | 12.09±0.04 | 10.61±0.10 | 9.92±0.07 | 9.59±0.08 | 8.91±0.12 | 11.06±0.06 | 10.50±0.07 | 10.64±0.11 | 9.43±0.10 | 9.24±0.11 | 9.48±0.12 | 11.58±0.04 |
| Mongolian-A | 12.25±0.05 | 10.96±0.09 | 10.14±0.07 | 9.52±0.08 | 9.12±0.09 | 10.92±0.09 | 10.47±0.06 | 10.31±0.11 | 9.01±0.09 | 9.42±0.08 | 8.89±0.11 | 11.66±0.04 |
| Mongolian-U | 12.31±0.07 | 11.07±0.16 | 10.26±0.11 | 9.22±0.11 | 8.90±0.12 | 10.59±0.12 | 10.54±0.08 | 10.07±0.19 | 8.89±0.13 | 9.17±0.15 | 8.85±0.17 | 11.64±0.08 |
| Mongolian-R | 12.20±0.06 | 10.86±0.09 | 10.04±0.08 | 9.77±0.08 | 9.32±0.11 | 11.20±0.10 | 10.42±0.08 | 10.51±0.11 | 9.11±0.12 | 9.64±0.06 | 8.93±0.15 | 11.68±0.05 |
| Kazakh-A | 12.31±0.05 | 10.72±0.12 | 10.18±0.11 | 9.43±0.12 | 8.96±0.19 | 11.57±0.10 | 10.52±0.08 | 10.33±0.17 | 8.97±0.14 | 10.15±0.13 | 8.89±0.13 | 11.86±0.06 |
| Kazakh-U | 12.34±0.07 | 10.80±0.19 | 10.06±0.13 | 9.43±0.14 | 8.83±0.23 | 11.65±0.13 | 10.62±0.09 | 10.48±0.21 | 9.16±0.17 | 10.08±0.15 | 8.90±0.13 | 11.93±0.07 |
| Kazakh-R | 12.27±0.09 | 10.60±0.14 | 10.34±0.17 | 9.42±0.22 | 9.13±0.35 | 11.46±0.14 | 10.38±0.12 | 10.12±0.29 | 8.70±0.22 | 10.24±0.23 | 8.87±0.26 | 11.75±0.11 |
| Han-A | 12.24±0.04 | 11.03±0.07 | 10.56±0.06 | 8.74±0.06 | 8.26±0.07 | 11.21±0.06 | 10.39±0.04 | 9.78±0.07 | 9.21±0.06 | 9.56±0.07 | 8.98±0.09 | 11.78±0.04 |
| Han-U | 12.18±0.05 | 10.97±0.11 | 10.48±0.08 | 8.77±0.09 | 8.32±0.11 | 11.16±0.09 | 10.32±0.05 | 9.88±0.10 | 9.22±0.08 | 9.49±0.11 | 9.01±0.12 | 11.74±0.06 |
| Han-R | 12.31±0.05 | 11.09±0.09 | 10.65±0.08 | 8.71±0.09 | 8.20±0.08 | 11.27±0.09 | 10.47±0.05 | 9.68±0.11 | 9.20±0.09 | 9.64±0.10 | 8.96±0.13 | 11.81±0.06 |
| Bai-A | 12.32±0.06 | 10.80±0.09 | 10.30±0.09 | 9.61±0.06 | 8.38±0.08 | 11.51±0.09 | 10.41±0.05 | 10.34±0.11 | 8.68±0.13 | 10.16±0.09 | 8.76±0.09 | 11.89±0.06 |
| Bai-U | 12.22±0.09 | 10.89±0.14 | 10.38±0.16 | 9.51±0.10 | 8.31±0.15 | 11.29±0.15 | 10.41±0.08 | 10.38±0.22 | 8.39±0.20 | 9.98±0.14 | 8.51±0.13 | 11.76±0.10 |
| Bai-R | 12.36±0.07 | 10.76±0.12 | 10.27±0.10 | 9.66±0.08 | 8.42±0.09 | 11.61±0.12 | 10.41±0.06 | 10.32±0.12 | 8.82±0.17 | 10.25±0.12 | 8.88±0.11 | 11.95±0.07 |

Remarks: Bacterial amounts are expressed in Log_10_ copy number of 16S rRNA per gram of fecal sample (mean ± S.E.M.). The mean ± S.E.M value calculated from all, only the urban-dwelling and only the rural-dwelling individuals are denoted by ‘A’, ‘U’ and ‘R’, respectively. Pairwise Mann-Whitney test was performed to compare between sample groups. The generated Bonferroni-corrected p-values between each sample pair are listed in Table S3.
