# Supplementary material for: Characterization of Fecal Microbiota across Seven Chinese Ethnic Groups by Quantitative Polymerase Chain Reaction
Source: PLoS One. 2014 Apr 3;9(4):e93631. doi: 10.1371/journal.pone.0093631 (PMC3974763; doi:10.1371/journal.pone.0093631)
Supplement: Table S3 — Bonferroni-corrected p-values generated by pairwise Mann-Whitney test comparing bacterial amounts of fecal samples from 7 ethnic groups. (DOC) [file pone.0093631.s005.doc]

Table S3. **Bonferroni-corrected p-values generated by pairwise Mann-Whitney test comparing bacterial amounts of fecal samples from 7 ethnic groups**

| **All bacteria** | Z | U | T | M | K | H | ***Clostridium coccoides* group** | Z | U | T | M | K | H | ***Clostridium leptum* group** | Z | U | T | M | K | H |
| --- | --- | --- | --- | --- | --- | --- | --- | --- | --- | --- | --- | --- | --- | --- | --- | --- | --- | --- | --- | --- |
| U | 1.0000 | -- | -- | -- | -- | -- | U | 1.0000 | -- | -- | -- | -- | -- | U | **0.0017** | -- | -- | -- | -- | -- |
| T | **0.0140** | 1.0000 | -- | -- | -- | -- | T | 1.0000 | 1.0000 | -- | -- | -- | -- | T | **0.0000** | 1.0000 | -- | -- | -- | -- |
| M | 1.0000 | 1.0000 | 0.0644 | -- | -- | -- | M | 0.7113 | 0.2533 | 0.1461 | -- | -- | -- | M | **0.0001** | 1.0000 | 1.0000 | -- | -- | -- |
| K | 1.0000 | 1.0000 | **0.0108** | 1.0000 | -- | -- | K | 1.0000 | 1.0000 | 1.0000 | 1.0000 | -- | -- | K | **0.0271** | 1.0000 | 1.0000 | 1.0000 | -- | -- |
| H | 1.0000 | 1.0000 | 0.3700 | 1.0000 | 1.0000 | -- | H | 0.0840 | 0.0573 | **0.0104** | 1.0000 | 1.0000 | -- | H | 1.0000 | **0.0032** | **0.0000** | **0.0002** | 0.1018 | -- |
| B | 1.0000 | 1.0000 | 0.0969 | 1.0000 | 1.0000 | 1.0000 | B | 1.0000 | 1.0000 | 1.0000 | 1.0000 | 1.0000 | 1.0000 | B | **0.0317** | 1.0000 | 0.0678 | 1.0000 | 1.0000 | 0.2263 |
| ***Clostridium perfringens* group** | Z | U | T | M | K | H | ***Lactobacillus* genus** | Z | U | T | M | K | H | ***Bacteroides fragilis* group** | Z | U | T | M | K | H |
| U | **0.0001** | -- | -- | -- | -- | -- | U | **0.0033** | -- | -- | -- | -- | -- | U | 1.0000 | -- | -- | -- | -- | -- |
| T | **0.0008** | 0.2390 | -- | -- | -- | -- | T | **0.0001** | 1.0000 | -- | -- | -- | -- | T | **0.0020** | 0.9196 | -- | -- | -- | -- |
| M | **0.0432** | 0.1039 | 1.0000 | -- | -- | -- | M | **0.0000** | 1.0000 | 0.4212 | -- | -- | -- | M | **0.0001** | 0.1805 | 1.0000 | -- | -- | -- |
| K | 1.0000 | **0.0377** | 1.0000 | 1.0000 | -- | -- | K | **0.0078** | 1.0000 | 1.0000 | 1.0000 | -- | -- | K | 1.0000 | 1.0000 | **0.0022** | **0.0015** | -- | -- |
| H | **0.0174** | **0.0000** | **0.0000** | **0.0000** | **0.0008** | -- | H | 1.0000 | **0.0242** | **0.0003** | **0.0000** | 0.0773 | -- | H | **0.0245** | 1.0000 | 1.0000 | 0.3714 | 0.1653 | -- |
| B | **0.0026** | 0.1019 | 1.0000 | 1.0000 | 1.0000 | **0.0000** | B | 0.5896 | 0.1643 | **0.0105** | **0.0000** | 0.3393 | 1.0000 | B | 1.0000 | 1.0000 | 0.0511 | **0.0019** | 1.0000 | 0.3123 |
| ***Prevotella* genus** | Z | U | T | M | K | H | ***Bifidobacterium* genus** | Z | U | T | M | K | H | ***Atopobium* cluster** | Z | U | T | M | K | H |
| U | 1.0000 | -- | -- | -- | -- | -- | U | **0.0005** | -- | -- | -- | -- | -- | U | 1.0000 | -- | -- | -- | -- | -- |
| T | 1.0000 | 1.0000 | -- | -- | -- | -- | T | **0.0000** | 1.0000 | -- | -- | -- | -- | T | 1.0000 | 1.0000 | -- | -- | -- | -- |
| M | 1.0000 | 1.0000 | 1.0000 | -- | -- | -- | M | **0.0000** | 1.0000 | 1.0000 | -- | -- | -- | M | 1.0000 | 1.0000 | **0.0142** | -- | -- | -- |
| K | 1.0000 | 1.0000 | 1.0000 | 1.0000 | -- | -- | K | **0.0015** | 1.0000 | 1.0000 | 1.0000 | -- | -- | K | 1.0000 | 0.8921 | 0.0707 | 1.0000 | -- | -- |
| H | 1.0000 | 0.6678 | 1.0000 | 1.0000 | 1.0000 | -- | H | 0.2383 | 0.1785 | **0.0000** | **0.0027** | 0.0773 | -- | H | 1.0000 | 1.0000 | 0.4536 | 1.0000 | 1.0000 | -- |
| B | 1.0000 | 1.0000 | 1.0000 | 1.0000 | 1.0000 | 1.0000 | B | **0.0000** | 1.0000 | 1.0000 | 1.0000 | 1.0000 | **0.0003** | B | **0.0275** | 0.0742 | **0.0003** | 0.9392 | 1.0000 | **0.0087** |
| ***Enterobacteriaceae* family** | Z | U | T | M | K | H | ***Desulfovibrio* genus** | Z | U | T | M | K | H | **Sum of the 10 bacterial groups** | Z | U | T | M | K | H |
| U | 1.0000 | -- | -- | -- | -- | -- | U | 1.0000 | -- | -- | -- | -- | -- | U | 1.0000 | -- | -- | -- | -- | -- |
| T | **0.0000** | **0.0000** | -- | -- | -- | -- | T | 1.0000 | 0.2809 | -- | -- | -- | -- | T | **0.0041** | 1.0000 | -- | -- | -- | -- |
| M | **0.0001** | **0.0007** | 1.0000 | -- | -- | -- | M | 0.3723 | 1.0000 | **0.0423** | -- | -- | -- | M | 0.2196 | 1.0000 | 1.0000 | -- | -- | -- |
| K | 1.0000 | 1.0000 | **0.0000** | **0.0014** | -- | -- | K | 0.2532 | 1.0000 | 0.0618 | 1.0000 | -- | -- | K | 1.0000 | 1.0000 | **0.0011** | 0.1819 | -- | -- |
| H | **0.0021** | **0.0186** | 0.0989 | 1.0000 | **0.0253** | -- | H | 0.5651 | 1.0000 | 0.0667 | 1.0000 | 1.0000 | -- | H | 1.0000 | 1.0000 | 0.1034 | 1.0000 | 1.0000 | -- |
| B | 1.0000 | 1.0000 | **0.0000** | **0.0000** | 1.0000 | **0.0008** | B | **0.0011** | 1.0000 | **0.0001** | 1.0000 | 1.0000 | 0.3652 | B | 1.0000 | 1.0000 | **0.0018** | 0.1725 | 1.0000 | 1.0000 |

Z: Zhuang; U: Uyghur; T: Tibetan; M: Mongolian; K: Kazakh; H: Han; B: Bai; p-value < 0.05 is considered statistically significant and is listed in bold font.
