# Supplementary material for: Characterization of Fecal Microbiota across Seven Chinese Ethnic Groups by Quantitative Polymerase Chain Reaction
Source: PLoS One. 2014 Apr 3;9(4):e93631. doi: 10.1371/journal.pone.0093631 (PMC3974763; doi:10.1371/journal.pone.0093631)
Supplement: Table S4 — Bacterial amounts of the 91 fecal samples of the Han subjects. (DOC) [file pone.0093631.s006.doc]

Table S4. **Bacterial amounts of the 91 fecal samples of the Han subjects**

|  |  | *Firmicutes* | | | | *Bacteroidetes* | | *Actinobacteria* | | *Proteobacteria* | |  |
| --- | --- | --- | --- | --- | --- | --- | --- | --- | --- | --- | --- | --- |
|  | All bacteria | *Clostridium coccoides* group | *Clostridium leptum* group | *Clostridium perfringens* group | *Lactobacillus* genus | *Bacteroides fragilis* group | *Prevotella* genus | *Bifidobacterium* genus | *Atopobium* cluster | *Enterobacteriaceae* family | *Desulfovibrio* genus | Sum of the 10 bacterial groups |
| Heilongjiang | 12.23±0.06 | 11.25±0.14 | 10.48±0.09 | 9.21±0.09 | 8.18±0.13 | 10.96±0.09 | 10.33±0.06 | 9.63±0.10 | 9.30±0.10 | 9.75±0.12 | 8.78±0.13 | 11.76±0.08 |
| Henan | 12.15±0.06 | 10.73±0.10 | 10.53±0.10 | 8.74±0.09 | 8.25±0.14 | 11.35±0.12 | 10.41±0.08 | 9.73±0.18 | 9.18±0.10 | 9.29±0.15 | 8.63±0.21 | 11.70±0.08 |
| Sichuan | 12.42±0.11 | 11.04±0.18 | 10.56±0.17 | 8.45±0.09 | 8.39±0.16 | 11.56±0.16 | 10.51±0.07 | 9.96±0.19 | 9.07±0.18 | 9.91±0.15 | 9.21±0.19 | 11.99±0.12 |
| Jiangsu | 12.21±0.06 | 11.02±0.12 | 10.73±0.13 | 8.32±0.13 | 8.28±0.12 | 11.13±0.12 | 10.35±0.08 | 9.90±0.13 | 9.22±0.10 | 9.32±0.16 | 9.48±0.10 | 11.71±0.07 |

Remarks: Bacterial amounts are expressed in Log_10_ copy number of 16S rRNA per gram of fecal sample (mean ± S.E.M.). Pairwise Mann-Whitney test was used to compare between sample groups. The generated Bonferroni-corrected p-values of sample pairs showing significant difference are listed in Table 4.
