# Supplementary material for: Characterization of Fecal Microbiota across Seven Chinese Ethnic Groups by Quantitative Polymerase Chain Reaction
Source: PLoS One. 2014 Apr 3;9(4):e93631. doi: 10.1371/journal.pone.0093631 (PMC3974763; doi:10.1371/journal.pone.0093631)
Supplement: Table S5 — Firmicutes / Bacteroidetes ( F/B ) ratio of different ethnic groups. (DOC) [file pone.0093631.s007.doc]

### Table S5. *Firmicutes*/*Bacteroidetes* (*F/B*) ratio of different ethnic groups

|  | Zhuang | Uyghur | Tibetan | Mongolian | Kazakh | Han | Bai |
| --- | --- | --- | --- | --- | --- | --- | --- |
| Mean *F*/*B* ratio  ± SEM | 0.6±0.11 | 0.75±0.26 | 1.08±0.35 | 3.12±0.88 | 0.56±0.16 | 4.03±1.03 | 1.07±0.24 |
|  |  |  |  |  |  |  |  |
| P-values (with Bonferroni correction) generated from pairwise Mann-Whitney test | | | | | | |  |
|  | Zhuang | Uyghur | Tibetan | Mongolian | Kazakh | Han |  |
| Uyghur | 1.0000 | -- | -- | -- | -- | -- |  |
| Tibetan | 1.0000 | 1.0000 | -- | -- | -- | -- |  |
| Mongolian | **0.0027** | 0.1270 | 0.0831 | -- | -- | -- |  |
| Kazakh | 1.0000 | 1.0000 | 1.0000 | **0.0321** | -- | -- |  |
| Han | **0.0005** | 0.0904 | **0.0354** | 1.0000 | **0.0139** | -- |  |
| Bai | 1.0000 | 1.0000 | 1.0000 | 0.0918 | 1.0000 | **0.0465** |  |

### p < 0.05 is considered as significantly different between sample pairs (listed in bold font).
